# Type B Lactic Acidosis: Diagnosis, Etiology and Treatment – An Educational Review

**DOI:** 10.14740/cr2265

**Published:** 2026-08-31

**Authors:** Juan A. Cardenas Fimbres, Jose M. Cardenas Fimbres, Claire Stewart, Joseph D. Tobias

**Affiliations:** a Division of Pediatric Critical Care Medicine, Department of Pediatrics, Nationwide Children’s Hospital and The Ohio State University College of Medicine, Columbus, OH 43205, USA; b Division of Pediatric Critical Care Medicine, Department of Pediatrics, University of Florida College of Medicine, Gainesville, FL, USA; c Department of Anesthesiology and Pain Medicine, Nationwide Children’s Hospital, Columbus, OH, USA; d Department of Anesthesiology and Pain Medicine, The Ohio State University College of Medicine, Columbus, OH, USA

**Keywords:** Lactic acidosis, Anaerobic metabolism, Type B lactic acidosis, Glycolysis, Hypoxia

## Abstract

Lactate elevation is commonly interpreted as a marker of tissue hypoxia; however, hyperlactatemia may occur despite adequate oxygen delivery and reflect diverse abnormalities in lactate production or clearance. Type B lactic acidosis is traditionally classified into three categories: underlying comorbid diseases, medication- and toxin-induced causes, and inborn errors of metabolism, making its recognition and management challenging. D-lactic acidosis represents a distinct disorder of lactate metabolism that may be overlooked by standard lactate assays. This educational review summarizes the pathophysiology and classification of type B lactic acidosis, reviews its major etiologies, and discusses D-lactic acidosis and hyperlactatemia during mechanical circulatory support. We propose a practical diagnostic algorithm for unexplained hyperlactatemia and review current therapeutic strategies, emphasizing treatment of the underlying cause. A structured approach may facilitate recognition of the mechanisms contributing to hyperlactatemia and guide targeted management, particularly in critically ill patients.

## Introduction

**Figure 1:**
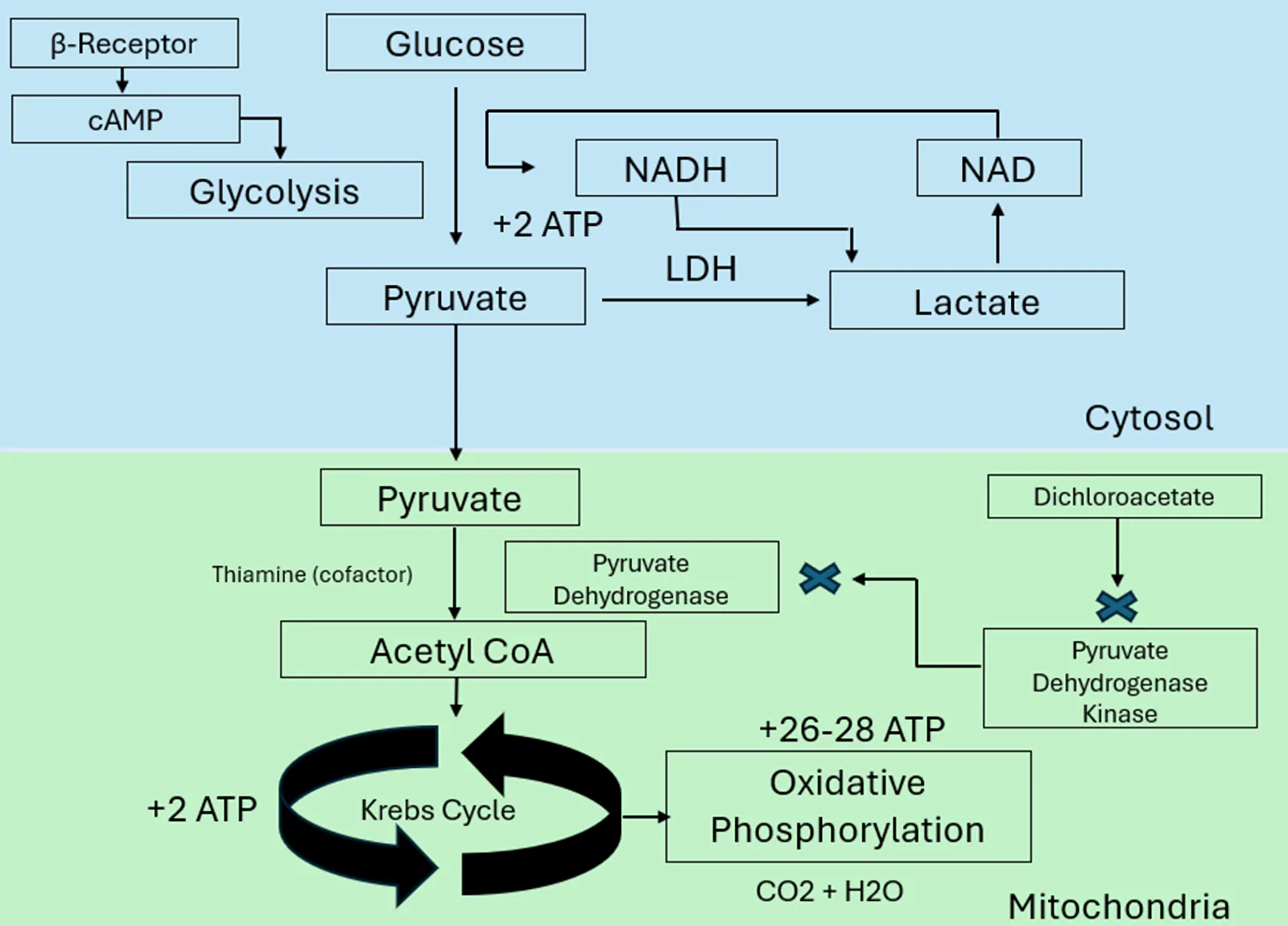
Normal aerobic pathway and anaerobic shift to lactate. ATP: adenosine triphosphate; CO_2_: carbon dioxide; LDH: lactate dehydrogenase; NADH: nicotinamide adenine dinucleotide (reduced form); NAD: nicotinamide adenine dinucleotide; cAMP: cyclic adenosine monophosphate.

Under conditions of adequate oxygen availability, the complete oxidative metabolism of one molecule of glucose through glycolysis, the citric acid cycle (commonly referred to as the Krebs cycle or the tricarboxylic acid cycle) and oxidative phosphorylation yields approximately 30–32 molecules of adenosine triphosphate (ATP), with the majority being generated during oxidative phosphorylation [1]. This process begins with glycolysis, in which glucose is converted to a three-carbon fragment, pyruvate, in the cytosol [2]. Pyruvate then enters the mitochondria, where it is converted into acetyl coenzyme A through a three-step process (pyruvate oxidation) governed by the enzyme, pyruvate dehydrogenase (PDH). This step is functionally oxygen-dependent, as oxidative phosphorylation is required to regenerate nicotinamide adenine dinucleotide (NAD^+^) and sustain PDH flux. Acetyl coenzyme A enters the citric acid cycle ultimately leading to complete oxidation into carbon dioxide and water [2]. Under normal physiological conditions, a small amount of pyruvate is also converted to lactate, even in the presence of adequate oxygen, particularly in tissues with high glycolytic rates such as red blood cells and skeletal muscle [3]. This process is facilitated by the enzyme lactate dehydrogenase. Under normal physiological conditions, lactate is converted back to glucose in the liver in the Cori cycle. However, in the setting of tissue hypoxia, whether due to local or systemic hypoperfusion, impaired oxygen delivery, or mitochondrial dysfunction, pyruvate is increasingly diverted away from mitochondrial oxidation and toward anaerobic metabolism [4]. This shift enhances lactate production and significantly reduces energy yield to two ATP molecules per glucose. However, it also regenerates NAD^+^, which is essential to sustain glycolysis (Fig. 1) [4].

**Figure 2:**
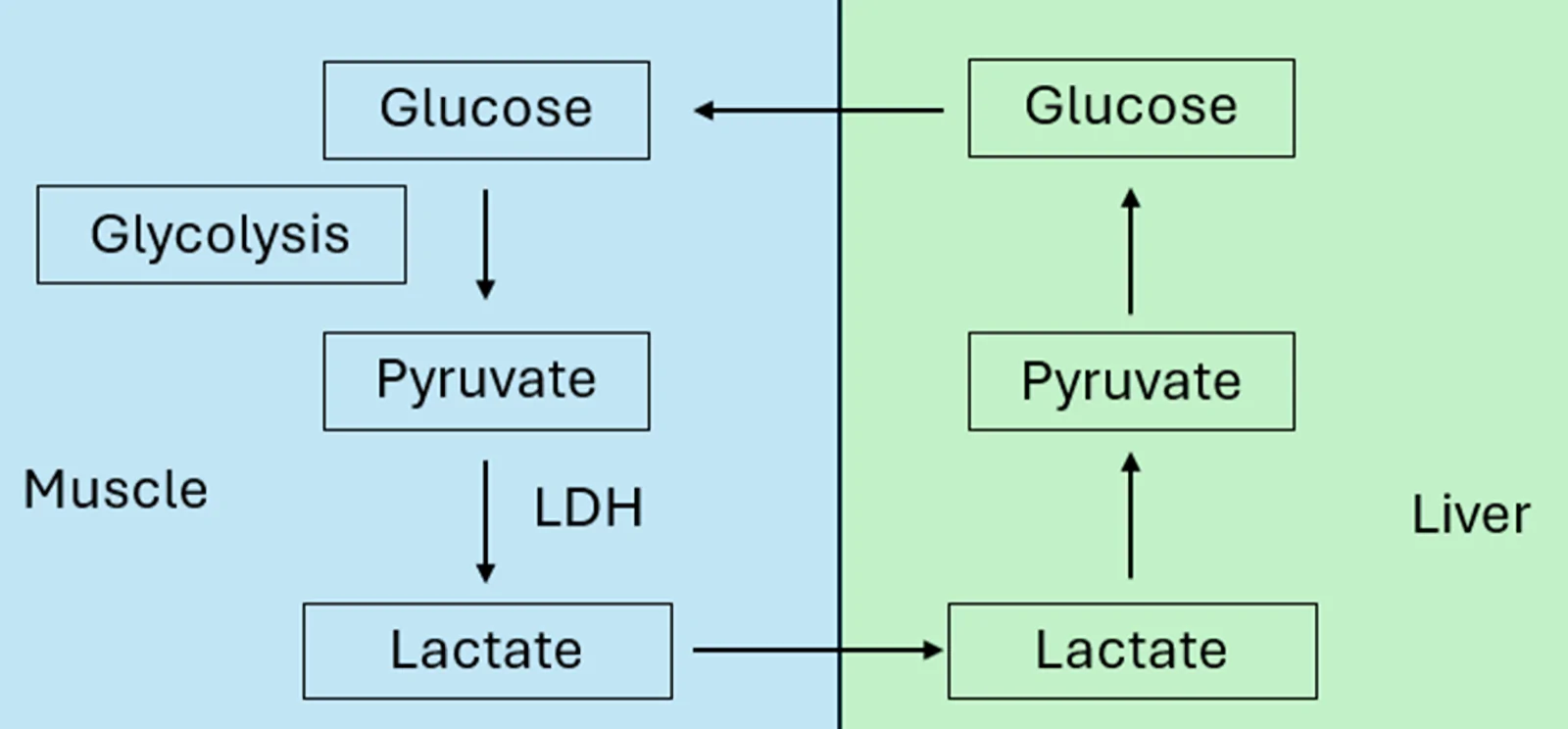
Cori cycle. LDH: lactate dehydrogenase.

Lactate, produced either during normal metabolic processes or during anaerobic conditions, can be converted back to pyruvate in the liver and kidneys, and further into glucose through gluconeogenesis, a process known as the Cori cycle (Fig. 2) [5]. Thus, lactate homeostasis depends not only on its production, but also on its metabolism, primarily by the liver, making hepatic function essential for the normal processing of lactate. When lactate production exceeds metabolism, whether due to excessive production or inadequate removal, it accumulates in the body, causing lactic acidosis.

Importantly, measured lactate may be spuriously elevated by pre-analytical factors, as continued *in vitro* glycolysis increases lactate over time, an effect accelerated at room temperature and minimized by rapid analysis or cooling. Routine tourniquet use does not meaningfully affect lactate levels, though prolonged application may modestly increase its concentration via localized anaerobic metabolism [6, 7].

The accumulation of lactate in low flow states and sepsis correlates with morbidity and mortality risk [8]. A prospective adult study conducted over 5 years across 10 tertiary care intensive care units involving 126 patients found that mortality reached up to 80% in individuals with lactate levels ≥ 5 mmol/L (normal range 0.5–1.8 mmol/L) accompanied by metabolic acidosis (defined as pH < 7.35 or base deficit > 6 mmol/L) [9, 10]. A retrospective study of 3,325 adult patients in a critical care setting found that lactate ≥ 4 mmol/L was strongly associated with a higher 3-day, 30-day, and 1-year mortality, particularly in sepsis [11]. A meta-analysis of 15 studies examined lactate clearance in critically ill patients, showing that higher lactate clearance is associated with improved survival and supporting its role as a valuable prognostic marker [12]. These findings highlight that elevated lactate, even high-normal values, are powerful independent predictors of adverse outcomes in critically ill patients, a relationship supported by multiple other studies demonstrating the correlation between lactic acidosis and morbidity and mortality. Moreover, because increased lactate clearance is associated with improved survival, these observations provide a rationale for targeting interventions that promote lactate reduction as part of the management of critically ill patients.

Lactic acidosis is broadly categorized into two types: type A and type B. Type A lactic acidosis, the most prevalent form, results from tissue hypoxia and is commonly observed in clinical shock conditions (cardiogenic, distributive, hypovolemic), as well as acute systemic hypoxemia [4]. In contrast, type B lactic acidosis occurs in the absence of overt tissue hypoxia and may be associated with mitochondrial dysfunction, specific medications, oncologic diseases, or inborn errors of metabolism [13]. This educational review discusses the various subtypes of type B lactic acidosis, focusing on their underlying mechanisms and clinical implications. Additionally, we review D-lactic acidosis as a distinct disorder of lactate metabolism, examine lactic acidosis during extracorporeal life support, and present a practical diagnostic approach and therapeutic interventions based on the underlying etiology.

## Materials and Methods

This article is a narrative review of the literature. Relevant publications were identified through searches of PubMed, Scopus, and Google Scholar using the terms lactic acidosis, anaerobic glycolysis, and pyruvate metabolism. The search terms were used individually and in combination. Additional references were identified through manual review of the bibliographies of selected articles. Articles relevant to the pathophysiology, diagnosis, and management of type B lactic acidosis were included at the authors’ discretion.

## Discussion

There are two different types of lactate based on their optical parameters including the isomers: L-lactate and D-lactate [14]. L-lactate is the predominant form produced by human cells during glycolysis and is the isomer routinely measured in clinical laboratories, while D-lactate is not routinely included in standard lactate assays [4]. L-lactate plays a central role in assessing tissue perfusion and metabolic function, particularly in critical care settings. D-lactate, on the other hand, is produced in small amounts via alternative pathways or by certain gut bacteria and is not detected by standard lactate assays [14]. While rare and not generally of clinical significance, D-lactate has specific clinical relevance and will be addressed later in this review.

As noted above, lactic acidosis is traditionally classified into two primary categories. Type A lactic acidosis arises in the context of tissue hypoperfusion or hypoxia, reflecting impaired oxygen delivery or utilization [15]. In contrast, type B lactic acidosis occurs despite adequate tissue perfusion and oxygenation [16]. Due to its heterogeneous nature, type B has been further subclassified into three distinct categories: (1) comorbid diseases, often due to hepatic or renal dysfunction; (2) medication and toxin induced; and (3) lactic acidosis secondary to inborn errors of metabolism, including mitochondrial disorders (Table 1) [16, 17]. Two additional clinically relevant scenarios are addressed separately: D-lactic acidosis, a distinct disorder of lactate metabolism, and hyperlactatemia during mechanical circulatory support, where type A and type B mechanisms may coexist.

**Table 1:** Etiologies of Type B Lactic Acidosis

Comorbid disease	Medication and toxin induced	Inborn errors of metabolism
Hepatic dysfunction	Albuterol	Pyruvate carboxylase deficiency
DKA (methylglyoxal pathway)	Epinephrine	Mitochondrial myopathies
Warburg effect	Theophylline	Glycogen storage diseases
Thiamine deficiency	Corticosteroids	Long-chain fatty acid β-oxidation disorders
	NRTIs	
	Propofol	
	Ethanol and methanol	
	Cyanide	
	Salicylate	

DKA: diabetic ketoacidosis; NRTIs: nucleoside-reverse transcriptase inhibitor.

### Comorbid diseases and type B lactic acidosis

#### Hepatic and renal dysfunction

Lactate homeostasis is primarily maintained through hepatic and renal clearance mechanisms. The liver plays the predominant role, accounting for approximately 50–70% of systemic lactate clearance via gluconeogenesis (Cori cycle) and oxidative metabolism [18]. In contrast, the kidneys contribute about 30–33%, mainly through lactate uptake and metabolism in the renal cortex [19]. Given the dominant role of the liver in lactate elimination, hepatic dysfunction or liver failure can significantly impair lactate clearance and is a more clinically relevant contributor to the development of type B lactic acidosis than renal impairment.

#### Infections including sepsis

Sepsis is a well-established cause of tissue hypoperfusion and a prototypical driver of type A lactic acidosis [15]. However, it may also result in mixed lactic acidosis (types A and B), or predominant type B lactic acidosis through non-hypoxic metabolic mechanisms. Hepatic dysfunction, frequently observed in sepsis due to direct inflammatory injury, hypoxic hepatitis, or cholestasis, compromises lactate clearance and contributes to systemic lactate accumulation [20]. Additionally, the profound inflammatory response seen in sepsis upregulates aerobic glycolysis and impairs PDH activity, shifting pyruvate metabolism toward lactate production even in the presence of adequate oxygen delivery and tissue oxygenation [21]. The situation is further exacerbated by the use of exogenous catecholamines, such as epinephrine, during sepsis and septic shock, which stimulate β_2_-adrenergic receptors and further enhance glycolytic flux [22]. Together, these mechanisms highlight the multifactorial nature of lactate elevation in sepsis, contributing to both type A and type B lactic acidosis.

#### Diabetic ketoacidosis (DKA)

A similar interplay between hypoperfusion and metabolic dysfunction can be observed in DKA. While type A lactic acidosis can develop in the setting of severe DKA due to hypovolemia-induced tissue hypoperfusion, the underlying pathophysiology is often more complex [23]. Notably, metformin, a common anti-hyperglycemic agent, has been associated with type B lactic acidosis, as it can impair mitochondrial oxidative phosphorylation and promote lactate accumulation independent of tissue hypoxia [24]. Additionally, a positive correlation between elevated glucose and lactate levels has been reported, suggesting an alternative metabolic pathway contributing to lactate production. Experimental studies in human skeletal muscle cells exposed to chronic hyperglycemia demonstrate that excess glucose increases glycolytic flux, resulting in increased lactate production despite preserved mitochondrial respiratory capacity, indicating impaired oxidative glucose utilization rather than hypoxia-driven lactate generation [25, 26]. In hyperglycemic states, increased flux through the glyoxalase system leads to the formation of methylglyoxal, a reactive glycolytic byproduct that is subsequently metabolized to lactate [27]. This mechanism provides additional insight into how lactate may accumulate in DKA beyond traditional hypoperfusion-related pathways, further illustrating the metabolic complexity underlying type B lactic acidosis.

#### Oncologic diseases and Warburg effect

Another notable source of lactic acidosis in the absence of tissue hypoxia is the Warburg effect. This phenomenon, commonly observed in malignant cells, describes a metabolic reprogramming in which cells preferentially convert glucose to lactate through anaerobic glycolysis, despite the presence of adequate oxygen [28]. This shift supports the high metabolic demands of rapidly proliferating cancer cells by facilitating biosynthetic precursor generation and maintaining redox balance [29]. As a result, persistent lactate accumulation can occur, contributing to type B lactic acidosis in patients with certain malignancies, particularly aggressive solid tumors such as brain, lung, colorectal, and breast cancers, even in the absence of overt organ dysfunction or hypoperfusion.

#### Thiamine deficiency

In addition to malignancy-related metabolic reprogramming, vitamin deficiencies, particularly thiamine (vitamin B1) deficiency, can contribute to type B lactic acidosis [13]. Thiamine serves as an essential cofactor for the PDH complex, which catalyzes the conversion of pyruvate to acetyl-CoA for entry into the Krebs cycle during aerobic metabolism (Fig. 1) [13, 30]. In the absence of sufficient thiamine, PDH activity is impaired, leading to the diversion of pyruvate toward lactate production via lactate dehydrogenase, even when oxygen is adequately available [31]. Clinically relevant causes of thiamine deficiency include chronic alcoholism, malabsorption and short gut syndromes, malignancy, poor nutritional intake, and functional deficiencies due to increased metabolic demands or altered absorption [32, 33].

### Medication and toxin-induced

#### Metformin

Although metformin-associated lactic acidosis (MALA) is relatively rare, it is potentially life-threatening. Type B lactic acidosis is caused by metformin-induced impairment of mitochondrial function in lactate-metabolizing tissues such as the liver and muscle [34]. This results in increased lactate production and reduced clearance. MALA risk is heightened by renal impairment, hepatic dysfunction, dehydration, sepsis, heart failure, hypoxia, or other conditions that limit metformin elimination or lactate metabolism [24].

#### Nucleoside reverse-transcriptase inhibitors (NRTIs)

The NRTIs, particularly older agents such as stavudine, didanosine, and zidovudine, can cause mitochondrial toxicity by inhibiting DNA polymerase γ, leading to mitochondrial DNA depletion and impaired oxidative phosphorylation [35]. This forces a shift toward anaerobic glycolysis despite adequate oxygen supply, increasing the nicotinamide adenine dinucleotide (NADH)/NAD^+^ ratio and promoting lactate accumulation [35]. While most patients maintain stable, low-grade lactate elevations, severe acidosis can occur abruptly without preceding hyperlactatemia [36].

#### Albuterol, epinephrine and dobutamine

Through β_2_ receptor activation, agents such as albuterol, epinephrine and, to a lesser extent, dobutamine increase intracellular cyclic adenosine monophosphate (AMP), stimulating glycogenolysis and glycolysis and accelerating ATP turnover via Na^+^/K^+^-ATPase activity (Fig. 1) [37]. This heightened aerobic glycolytic flux generates pyruvate at a rate that exceeds mitochondrial oxidative capacity, resulting in its preferential conversion to lactate, particularly in skeletal muscle, and contributing to the hyperlactatemia characteristic of type B lactic acidosis [37, 38]. Importantly, this process occurs despite adequate oxygen delivery, distinguishing it from the hypoxia-driven lactate accumulation of type A lactic acidosis which may be seen in sepsis and other conditions requiring the use of epinephrine. The clinical context in which these medications are used is therefore critical to interpreting lactate elevations.

For example, although albuterol, epinephrine and dobutamine are causes of type B lactic acidosis by stimulating aerobic glycolysis, high-dose therapy may also precipitate type A lactic acidosis in susceptible patients by increasing myocardial oxygen demand while reducing coronary perfusion. Coronary blood flow to the left ventricle occurs predominantly during diastole. Through β_2_-adrenergic receptor-mediated vasodilation, albuterol and dobutamine lowers diastolic blood pressure, thereby reducing coronary perfusion pressure [39]. Simultaneously, albuterol, epinephrine and dobutamine induced tachycardia increases myocardial oxygen consumption while shortening diastolic filling time, further limiting coronary perfusion [39]. The resulting imbalance between myocardial oxygen supply and demand may lead to myocardial ischemia, impaired cardiac output, tissue hypoperfusion, and secondary type A lactic acidosis [40, 41].

#### Adjunctive therapies in asthma

Other agents commonly used in asthma management, including theophylline, intravenous magnesium, and isoflurane, may affect hemodynamics and, in rare cases, cause sufficient hypotension to impair tissue perfusion, resulting in type A lactic acidosis [42–45]. Theophylline, a non-selective phosphodiesterase inhibitor, potentiates β-adrenergic effects by further increasing cyclic adenosine monophosphate (cAMP) levels [46]. Glucocorticoids, although not directly causing type B lactic acidosis, can upregulate β_2_-adrenergic receptors and enhance β-agonist–mediated lactate production, which is particularly relevant in asthma where corticosteroids are commonly administered [47]. While these medications are contributors to β_2_-adrenergic mediated type B lactic acidosis, the associated hemodynamic changes mean that type A lactic acidosis may also occur concurrently, so both mechanisms can coexist.

#### Propofol

Lactic acidosis, classically described as part of propofol infusion syndrome (PRIS), is a rare but often fulminant complication occurring primarily in children and in critically ill patients receiving high-dose or prolonged propofol infusions [48, 49]. The metabolic disturbance results from impaired mitochondrial uptake and oxidation of long-chain fatty acids, while the metabolism of medium- and short-chain fatty acids remains relatively preserved [50]. The resulting disruption of mitochondrial β-oxidation and oxidative phosphorylation leads to progressive accumulation of lactate, triglycerides and severe metabolic acidosis. The process can lead to progressive myocardial failure, arrhythmias, rhabdomyolysis and mortality from cardiac failure or metabolic derangements.

#### Ethanol, ethylene-glycol and methanol ingestion

The metabolism of ethanol by alcohol dehydrogenase to acetaldehyde and then further metabolism to acetate by aldehyde dehydrogenase converts NAD^+^ to NADH, increasing the NADH/NAD^+^ ratio, reducing the activity of PDH, and thereby favoring the reduction of pyruvate to L-lactate via lactate dehydrogenase [51]. This process can be further exacerbated by thiamine deficiency, commonly associated with chronic alcohol use which decreases PDH activity and enhances lactate production [13]. Methanol intoxication may also result in lactic acidosis; however, its mechanism is distinct and involves mitochondrial dysfunction through inhibition of respiratory chain complex IV [52].

#### Cyanide poisoning

Cyanide toxicity induces mitochondrial dysfunction through inhibition of cytochrome oxidase (complex IV) in the electron transport chain. This intoxication can occur via inhalation of smoke from fires, occupational exposure, or as a complication of prolonged sodium nitroprusside infusion [53].

#### Salicylate overdose

Salicylates stimulate the medullary respiratory center, causing respiratory alkalosis, while simultaneously uncoupling oxidative phosphorylation and inhibiting citric acid cycle enzymes, resulting in impaired ATP production and a metabolic shift toward glycolysis [54]. This metabolic derangement leads to increased production of lactate and ketoacids, contributing to an anion-gap metabolic acidosis that is independent of tissue hypoxia [54]. Salicylates are encountered in multiple forms, including acetylsalicylic acid tablets, methyl salicylate–containing topical preparations, bismuth subsalicylate, and salicylate rich foods and herbal products [55].

### Inborn errors of metabolism

#### Pyruvate carboxylase (PC) deficiency

PC deficiency is a rare autosomal recessive metabolic disorder characterized by impairment of the citric acid cycle, specifically the inability to convert pyruvate into oxaloacetate, a key intermediate of glucose metabolism [55]. This disruption leads to deficient ATP production which impacts high energy tissues (central nervous system, cardiac muscle, and skeletal muscle). Impairment of the citric acid cycle leads to the accumulation of pyruvate, which is subsequently converted to lactate, resulting in type B lactic acidosis. This pathologic process occurs despite the presence of adequate tissue oxygen [55]. Clinical severity varies, with some forms presenting in the neonatal period with severe metabolic acidosis, neurologic impairment, and early death, while milder forms may present later with intermittent and variable symptoms [30, 56].

#### Mitochondrial myopathies

Mitochondrial diseases are a group of inherited disorders primarily characterized by defects in oxidative phosphorylation, the main pathway for cellular energy production [57]. As these disorders affect primarily mitochondrial DNA, they follow the maternal lineage. Affecting approximately 1 in 5,000 individuals, these defects prevent cells from efficiently generating ATP through aerobic metabolism, even in the presence of adequate oxygen [58]. As a result, energy production shifts toward anaerobic glycolysis, leading to excessive lactate accumulation, a hallmark of type B lactic acidosis. This form is not due to hypoxia, but rather impaired cellular respiration (oxidative phosphorylation). Lactate levels may be chronically elevated or worsened during stress, illness, or fasting [59]. Common syndromes include mitochondrial encephalomyopathy, lactic acidosis, and stroke-like episodes (MELAS) and Leigh syndrome [60].

#### Glycogen storage diseases (GSD)

GSD type I (GSD I, von Gierke disease) causes lactic acidosis due to a deficiency in glucose-6-phosphatase (GSD Ia) or glucose-6-phosphate translocase (GSD Ib), which blocks the final step of glycogenolysis and gluconeogenesis [61]. As a result, glucose-6-phosphate accumulates and is shunted into glycolysis, leading to excessive pyruvate conversion to lactate [59]. This contributes to persistent hyperlactatemia and type B lactic acidosis, even in the absence of hypoperfusion [62]. In contrast, other GSDs (e.g., types III, VI, IX) generally do not cause significant lactic acidosis; mild elevations may occur only under fasting or metabolic stress conditions [62].

#### Long-chain fatty acid β-oxidation disorders

In long-chain fatty acid β-oxidation disorders, impaired mitochondrial fatty acid oxidation reduces generation of acetyl-CoA, NADH, and FADH_2_, leading to inadequate TCA cycle flux and oxidative phosphorylation [63]. Accumulation of long-chain acyl-CoA intermediates further disrupts mitochondrial energy metabolism, limiting pyruvate oxidation [63]. Consequently, pyruvate is preferentially reduced to lactate, resulting in type B lactic acidosis, particularly during fasting or metabolic stress, independent of tissue hypoxia.

### D-lactic acidosis

D-lactic acidosis is a rare form of lactic acidosis caused by accumulation of D-lactate, a stereoisomer poorly metabolized by humans. Although it occurs without tissue hypoxia and is considered a form of type B lactic acidosis, it is not included in the three traditional subtypes because it represents a distinct form of lactate metabolism disorder [14]. It is most often seen in patients with short bowel syndrome, where unabsorbed carbohydrates are fermented by colonic bacteria into D-lactate, which is then absorbed systemically [64]. Because standard lactate assays detect only L-lactate, D-lactic acidosis may be missed unless a dedicated D-lactate assay is specifically ordered, such as an enzymatic method using D-lactate dehydrogenase [4, 65]. Clinical manifestations include metabolic acidosis and neurologic symptoms such as confusion or encephalopathy [66].

### Hyperlactatemia during mechanical circulatory support

#### Lactic acidosis and cardiopulmonary bypass (CPB)

Hyperlactatemia during CPB occurs in a bimodal pattern. Early hyperlactatemia, which develops during CPB or within the first 4 h after CPB, is typically associated with inadequate oxygen delivery, as evidenced by a low cardiac index and reduced mixed venous oxygen saturation (SvO2). This pattern represents type A lactic acidosis and is associated with a worse prognosis [67].

In contrast, late-onset hyperlactatemia, occurring more than 4 h after CPB, is generally observed despite adequate global oxygen delivery and is associated with hyperglycemia and prolonged CPB duration [67]. Prolonged CPB is associated with a greater systemic inflammatory response and microcirculatory dysfunction, characterized by reduced perfused vessel density and increased microcirculatory heterogeneity, which may contribute to persistent postoperative hyperlactatemia [68]. Longer CPB duration is also associated with increased endogenous catecholamine release and greater use of exogenous catecholamines, which may promote accelerated aerobic glycolysis and contribute to lactate production [67]. More recent studies have additionally identified associations between postoperative hyperlactatemia, impaired microcirculatory blood flow, and reduced mitochondrial respiration following cardiovascular surgery with CPB [69]. Unlike early hyperlactatemia, late-onset hyperlactatemia has generally not been associated with increased mortality [67].

#### Lactic acidosis during extracorporeal life support

Hyperlactatemia during extracorporeal life support has multifactorial causes, and type B mechanisms should be considered after evaluating for inadequate systemic or regional perfusion and overt ischemia. Several mechanisms may contribute to persistent hyperlactatemia. First, hypocarbia resulting from excessive sweep gas flow may impair tissue oxygen unloading through a leftward shift of the oxyhemoglobin dissociation curve [70]. Second, occult ischemia should be considered, including mesenteric or other visceral organ thrombosis, acute limb ischemia, central nervous system injury or hemorrhage, and differential hypoxemia (Harlequin syndrome) in patients supported with venoarterial extracorporeal membrane oxygenation (VA-ECMO) [71, 72]. Third, impaired lactate clearance secondary to hepatic dysfunction or acute kidney injury may contribute to elevated lactate concentrations [71].

A practical diagnostic approach should focus on confirming adequate systemic and regional perfusion. This includes assessment of circulatory flow and cardiac output, evaluation of oxygen extraction, and investigation for regional or differential ischemia. Differential oxygenation can be assessed by comparing upper- and lower-body oxygenation using a right radial arterial catheter, pulse oximetry in the upper and lower extremities, cerebral near-infrared spectroscopy (NIRS), and echocardiography to evaluate left ventricular function and native cardiac output [71, 72]. When occult ischemia is suspected, appropriate imaging should be performed to evaluate for end-organ hypoperfusion, thrombosis, or hemorrhage [71, 72]. Depending on the clinical presentation, this may include computed tomography angiography, vascular ultrasound, abdominal imaging to assess mesenteric perfusion, neuroimaging for cerebrovascular injury or intracranial hemorrhage, and echocardiographic assessment for intracardiac thrombus or embolic phenomena [71, 72].

### Diagnosis

In clinical practice, the source of metabolic acidosis is not always readily identifiable. The anion gap is a useful diagnostic tool that helps distinguish high anion gap metabolic acidosis, often caused by accumulation of unmeasured anions such as lactate, from other metabolic acid-base disorders. The anion gap is based on the principle of electroneutrality, which requires balance between plasma cations and anions [73]. Because not all electrolytes are routinely measured, the anion gap estimates the difference between the major measured cation (sodium) and anions (chloride and bicarbonate). The normal anion gap is approximately 8–12 mEq/L [73].

Accumulation of measured anions, such as chloride in hydrochloric acid acidosis, does not increase the anion gap. In contrast, high anion gap metabolic acidosis results from accumulation of unmeasured anions, including lactate and other organic acids. Because albumin is the most abundant unmeasured plasma anion, variations in serum albumin concentration affect the measured anion gap [73]. Using a normal albumin concentration of 4 g/dL as the reference, the anion gap decreases by approximately 2.5 mEq/L for every 1 g/dL reduction in albumin; therefore, correction for hypoalbuminemia is necessary when interpreting the anion gap [73].

When evaluating a patient with lactic acidosis, it is essential to first rule out causes of type A lactic acidosis. A thorough clinical assessment, hemodynamic evaluation, and targeted laboratory and imaging studies are necessary to identify or exclude hypoxic mechanisms before attributing high lactate states to a type B (non-hypoxic) process. An evaluation of mixed venous oxygen concentration may be helpful in evaluating tissue oxygen delivery. Only after these causes have been excluded should the diagnostic workup focus on metabolic, toxic, or systemic disorders that impair lactate metabolism without overt tissue hypoxia, resulting in type B lactic acidosis.

Once type A lactic acidosis has been excluded, the lactate-to-pyruvate (L/P) ratio can be a useful next step to help provide additional insight into the diagnosis of non-hypoxic hyperlactatemia (type B). In states of tissue hypoxia, impaired oxidative phosphorylation leads to an accumulation of NADH relative to NAD^+^, shifting the redox balance and increasing the L/P ratio [74]. This reflects a shift toward anaerobic metabolism and is often associated with metabolic acidosis. Conversely, in non-hypoxic settings characteristic of type B lactic acidosis, including states of increased glycolytic activity or certain metabolic disorders, lactate may rise without a corresponding increase in the L/P ratio, which typically remains near the normal range (< 10:1), indicating preserved redox status and mitochondrial function [74]. In contrast, lactate increases disproportionately compared with pyruvate in type A lactic acidosis due to tissue hypoxia, leading to a markedly elevated L/P ratio [74]. Interpretation of the L/P ratio requires consideration of technical limitations, as pyruvate is chemically unstable and requires rapid specimen processing to ensure accurate results [75]. In addition, lactate and pyruvate measurements are not routinely available in many clinical laboratories, limiting their widespread use in the acute evaluation of lactic acidosis. Under normal conditions, plasma pyruvate concentrations are approximately 0.08–0.16 mmol/L, although values may vary depending on assay methodology and specimen handling [75].

**Figure 3:**
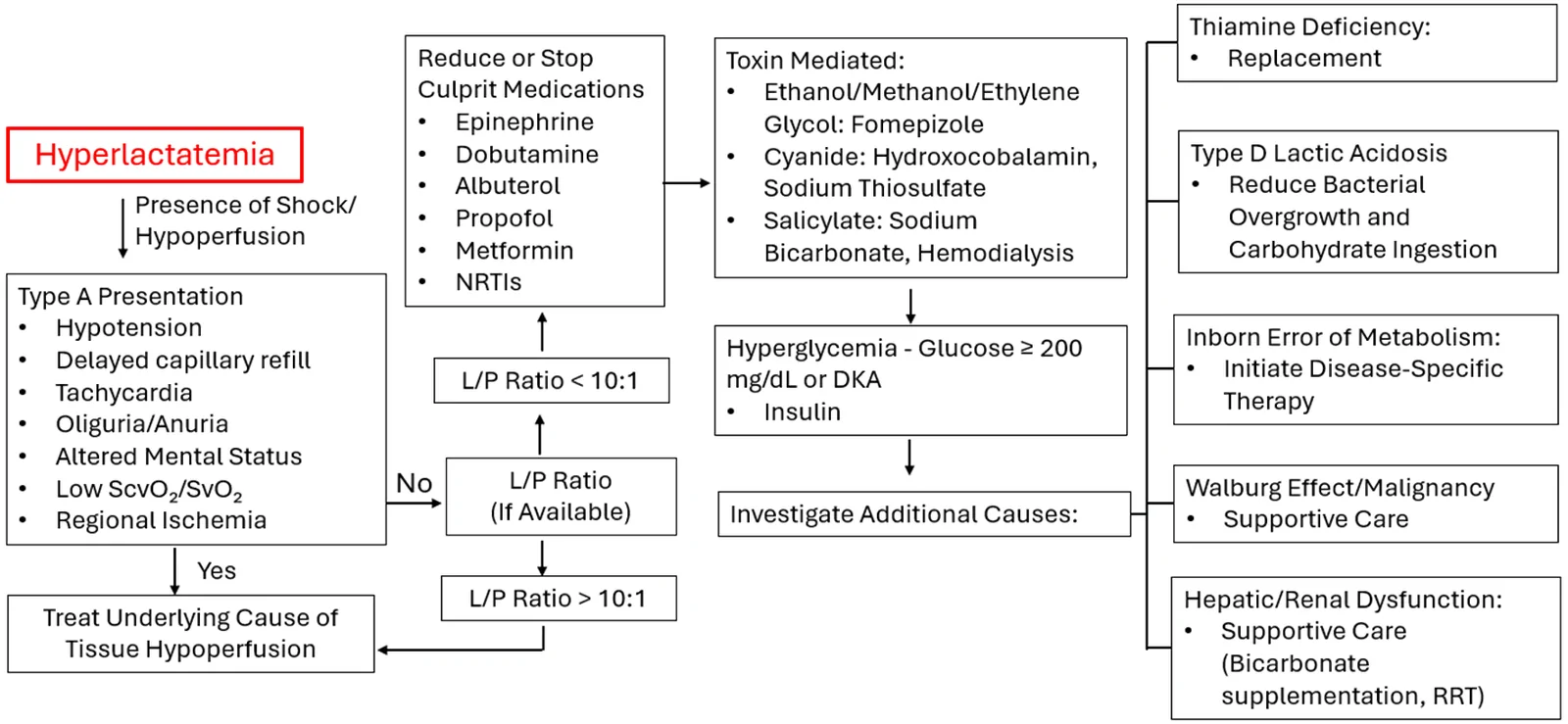
Diagnostic approach and management of type B lactic acidosis. Low ScvO_2_/SvO_2_: reduced oxygen delivery or increased oxygen extraction; L/P: lactate to pyruvate ratio; DKA: diabetic ketoacidosis; RRT: renal replacement therapy.

Because no standardized diagnostic algorithm exists for type B lactic acidosis, we propose a practical diagnostic approach that also incorporates general management principles (Fig. 3). The goal of this algorithm is to facilitate a systematic evaluation by prioritizing the identification of potentially reversible or intervenable causes. This proposed approach reflects the authors’ interpretation of the available evidence and is intended as a practical framework rather than a guideline-endorsed recommendation.

### Treatment strategies

Currently, there is no targeted treatment specifically for type B lactic acidosis, and management primarily involves identifying and addressing the underlying cause [76]. For practical purposes, management can be divided into three broad categories: (1) supportive management of severe acidemia while definitive treatment of the underlying cause is pursued; (2) discontinuation or dose reduction of offending agents, when clinically feasible; (3) targeted treatment of reversible or potentially intervenable causes.

#### Supportive management of severe acidemia

In instances where acidosis is severe enough to impair hemodynamic function, reduce myocardial contractility, increase the risk of arrhythmias, or diminish responsiveness to vasoactive agents, adjunctive buffering therapies may be used as a temporary supportive measure while the underlying cause is addressed [77, 78].

##### (1) Bicarbonate therapy

Bicarbonate therapy is one of the management options for metabolic acidosis but requires preserved pulmonary and circulatory function to enable effective elimination of the generated CO_2_ through ventilation. With repeated or high dosing, the high sodium of sodium bicarbonate content can increase plasma osmolality [79]. This increased ventilatory demand may exacerbate metabolic stress. As an alternative, acetate-containing fluids can serve as a bicarbonate precursor to help correct metabolic acidosis; unlike lactate-containing solutions such as lactated Ringer’s, their metabolism does not strictly depend on intact liver function [80]. Importantly, bicarbonate therapy is not routinely recommended, as clinical trials have not demonstrated a consistent improvement in mortality or clinical outcomes with its use [81–83].

##### (2) Renal replacement therapy (RRT)

RRT serves as an important intervention in managing metabolic acidosis [84]. By removing excess fluid and correcting hyperosmolality, RRT supports hemodynamic stability and acid–base balance, becoming particularly important when therapies with high sodium content or large infusion volumes increase osmolar load beyond the capacity of conventional management or physiologic compensatory mechanisms. RRT is an invasive procedure with associated risks that must be carefully considered [56].

##### (3) Dichloroacetate (DCA)

DCA acts by inhibiting PDH kinase, thereby maintaining it in its active, unphosphorylated state (Fig. 1) [56]. PDH kinase is a family of enzymes that inactivate PDH by phosphorylating it. The inhibition of PDH kinase increases the activity of PDH which shifts pyruvate metabolism away from lactate production toward conversion into acetyl-CoA, facilitating entry into the Krebs cycle and enhancing aerobic energy production [56]. DCA has been explored primarily as a therapeutic option in mitochondrial disorders and PDH complex deficiency, where impaired aerobic metabolism leads to lactic acidosis [85]. However, clinical efficacy remains incompletely established, and long-term administration is associated with adverse effects, notably peripheral neuropathy, which limits its widespread use [86].

#### Discontinuation or dose reduction of offending agents

After excluding causes of type A lactic acidosis, discontinuation of medications implicated in type B lactic acidosis should be considered when clinically feasible [87]. However, certain clinical scenarios require continuation of these therapies despite their potential contribution to lactate elevation. For example, in status asthmaticus, albuterol may be essential for respiratory management and cannot always be discontinued immediately; instead, dose reduction or adjustment of treatment frequency should be considered when clinically appropriate. Similarly, epinephrine may be necessary for inotropic or vasopressor support in critically ill patients, and although abrupt discontinuation may not be possible, dose reduction or substitution with alternative agents should be considered when hemodynamically feasible. Conversely, medications that are not essential for acute management or carry significant risk, such as propofol due to the potential for propofol infusion syndrome in children, should be discontinued when type B lactic acidosis is suspected [87].

#### Targeted treatment of reversible or potentially intervenable causes

When type B lactic acidosis results from toxin exposure or medication overdose, treatment should be directed toward the specific underlying cause. In cases of toxic alcohol ingestion, including methanol and ethylene glycol, fomepizole should be administered to inhibit alcohol dehydrogenase and prevent the formation of toxic metabolites [88]. In cyanide poisoning, such as from sodium nitroprusside toxicity or smoke inhalation, antidotal therapy with hydroxocobalamin should be considered. Hydroxocobalamin binds cyanide to form cyanocobalamin, which is subsequently excreted in the urine, thereby removing cyanide and relieving its inhibition of mitochondrial cytochrome c oxidase, allowing oxidative phosphorylation to resume. Sodium thiosulfate may also be used as an adjunctive or alternative antidote; it serves as a sulfur donor for rhodanese, facilitating the conversion of cyanide to thiocyanate, a less toxic metabolite that is subsequently eliminated renally [52]. In settings of excessive glycolytic flux associated with hyperglycemia or insulin deficiency, correction of the underlying metabolic disturbance with insulin may help reduce hyperlactatemia by restoring more physiologic glucose and pyruvate metabolism [25, 26].

## Conclusions

Type B lactic acidosis represents a diverse group of metabolic disorders in which lactate accumulates despite adequate tissue oxygen delivery. Unlike type A lactic acidosis, where lactate elevation reflects hypoxia and impaired perfusion, type B lactic acidosis arises from altered cellular metabolism, impaired clearance, or mitochondrial dysfunction. Recognition of this distinction is critical, as elevated lactate in non-hypoxic states does not uniformly indicate tissue ischemia and may lead to misinterpretation of illness severity or inappropriate escalation of resuscitative therapies.

A systematic diagnostic approach that first excludes hypoxic causes and then incorporates clinical context, medication exposure, organ function, and metabolic evaluation—including consideration of the lactate-to-pyruvate ratio—can help clarify the underlying mechanism of lactate elevation. Management remains largely supportive and etiology-driven, emphasizing correction of reversible contributors such as drug toxicity, nutritional deficiencies, and organ dysfunction rather than lactate normalization alone.

As lactate continues to be widely used as a biomarker of disease severity and prognosis, improved understanding of type B lactic acidosis is essential for accurate clinical interpretation and targeted management. Future research is needed to better define diagnostic tools, clarify the role of metabolic therapies, and develop tailored interventions for specific subtypes of type B lactic acidosis.

## Data Availability

All information discussed in this review was obtained from previously published sources cited in the reference list. No new data were generated or analyzed for this study.
